# Integrative Genomic Analyses of 1,145 Patient Samples Reveal New Biomarkers in Esophageal Squamous Cell Carcinoma

**DOI:** 10.3389/fmolb.2021.792779

**Published:** 2022-01-21

**Authors:** Binbin Zou, Dinghe Guo, Pengzhou Kong, Yanqiang Wang, Xiaolong Cheng, Yongping Cui

**Affiliations:** ^1^ Key Laboratory of Cellular Physiology of the Ministry of Education, Shanxi Medical University, Taiyuan, China; ^2^ Department of Pathology, Shanxi Medical University, Taiyuan, China; ^3^ Shenzhen Peking University-Hong Kong University of Science and Technology (PKU-HKUST) Medical Center, Peking University Shenzhen Hospital, Shenzhen, China

**Keywords:** esophageal squamous cell carcinoma, mutation, neoantigen, immunotherapy, KMT2D, bioinformactics

## Abstract

Due to the lack of effective diagnostic markers and therapeutic targets, esophageal squamous cell carcinoma (ESCC) shows a poor 5 years survival rate of less than 30%. To explore the potential therapeutic targets of ESCC, we integrated and reanalyzed the mutation data of WGS (whole genome sequencing) or WES (whole exome sequencing) from a total of 1,145 samples in 7 large ESCC cohorts, including 270 ESCC gene expression data. Two new mutation signatures and 20 driver genes were identified in our study. Among them, *AP3S1*, *MUC16*, and *RPS15* were reported for the first time. We also discovered that the *KMT2D* was associated with the multiple clinical characteristics of ESCC, and *KMT2D* knockdown cells showed enhanced cell migration and cell invasion. Furthermore, a few neoantigens were shared between ESCC patients. For ESCC, compared to TMB, neoantigen might be treated as a better immunotherapy biomarker. Our research expands the understanding of ESCC mutations and helps the identification of ESCC biomarkers, especially for immunotherapy biomarkers.

## Introduction

Esophageal cancer (EC) is the fourth most common cancer in China, with 375,000 annual deaths, 90% of which are esophageal squamous cell carcinoma (ESCC) (Chen et al., 2016). Due to the lack of effective diagnostic markers, most of ESCC patients are diagnosed at the advanced stages and are not suitable for surgical treatment. Due to the limited treatment methods, the 5 years survival rate of ESCC is less than 30% (Younes et al., 2002; Koshy et al., 2004). Therefore, it is imperative to explore new biomarkers and novel targets for ESCC diagnosis, prognosis, and clinical treatment.

In recent years, series of whole genome sequencing (WGS) and whole exome sequencing (WES) studies had been performed in ESCC (Lin et al., 2014; Song et al., 2014; Cao et al., 2015; Zhang et al., 2015; Hu et al., 2016; Qin et al., 2016; Sawada et al., 2016; Chang et al., 2017; Chen et al., 2017; Dai et al., 2017; Du et al., 2017; Liu et al., 2017; Nature, 2017; Dai et al., 2018; Yan et al., 2019; Yu et al., 2019; Cui et al., 2020; Dutta et al., 2020; He et al., 2020; Liu et al., 2020; Mangalaparthi et al., 2020). As a result of that, several ESCC driver genes had been identified. However, limitations in sample size and the algorithms used in driver gene identification still exist. More than half of the driver genes were identified in only one cohort, and few biomarkers were identified as especial immunotherapy-related biomarkers. At the same time, because of the lack of therapeutic targets for ESCC, targeted therapy treatment has not been improved significantly.

Here, we performed an integrative analysis using multiple datasets from 7 published cohorts, 270 ESCC gene expression data to explore potential biomarker especially immunotherapy biomarkers of ESCC. We found that the integration of data with a larger sample size is helpful in not only discovering new mutation signatures and new driver genes, but also identifying more potential biomarkers, especially low-frequency mutation genes.

## Methods

### Data Collection and Analysis

ESCC mutation data: The DNA mutation data was downloaded from the results of articles of 7 cohorts (Lin et al., 2014; Song et al., 2014; Zhang et al., 2015; Sawada et al., 2016; Chang et al., 2017; Nature, 2017; Cui et al., 2020), which provided whole mutation data, whole clinical information of sample and the sample number was bigger. All mutation results were uniformly converted into MAF format (https://docs.gdc.cancer.gov/Data/File_Formats/MAF_Format, Supplementary Table S2, Supplementary Table S3 was the clinical information of samples). ESCC gene expression data: 1) TCGA-ESCA gene expression data download from UCSC xena, different expression genes (DEGs) were identified by *t* test. 2) GSE53625 chip data download from NCBI GEO. Use blast alignment for each probe of the GSE53625 chip data against the hg19 reference genome. 100% alignment was required, and mismatches and indels were not allowed. For multiple probes aligned to the same gene, the expression value of the gene was the average of all probes. There were a total of 270 ESCC gene expression data.

### Mutation Signature Analysis

We used sigflow to analyze the mutation signature of the exon regions of all samples in SBS, DBS, ID types, and we also analyzed the whole genome of the 508 samples in SBS, DBS, ID types.

### TMB and Neoantigen Analysis

TMB calculation: total TMB (aTMB)=(SNV + indel)/30M, fTMB= (exclude nonsynonymous SNV + indel)/30 M.

HLA allele typing: Use optitype to identify HLA-A, HLA-B, HLA-C alleles on the 90 WES samples.

Neoantigen identified: First used maf2vcf (https://github.com/mskcc/vcf2maf) to convert the MAF to VCF, then used vep (McLaren et al., 2016) to annotate VCF referred to pvactools manual, next used pvactools to perform neoantigen analysis on samples. The BindLevel of the peptide with SB or WB as candidate neoantigen.

**Tumor neoantigen burden (TNB)**: Count the number of all candidate neoantigen in the sample.

**Tumor neoantigen score (TNS)**: The neoantigen score was the sum of the scores of all mutant peptides of the gene (Gscore).

$$\mathrm{Gsore}=\sum_{1}^{n}\frac{AAscore}{M},\mathrm{AAscore}=\{\begin{array}{c} \log_{2}(W/M)\;\\ 15 \end{array}$$

Where Gscore represented the neoantigen score of the gene, W represented the binding score of wild-type peptide with the HLA allele, M represented the binding score of the mutant peptide with the HLA allele, and AAscore represented the ratio of wild-type peptide binding score to mutant peptide binding score, if the mutant peptides did not have a corresponding wild-type peptide, such as mutant peptides produced by frameshift caused by indel, the ratio was 15.

### Driver Gene Analysis

MutSigCV, driverml, oncodrivefml, and oncodriveclutl were used to identify driver genes based on coding region mutation of 1145 ESCC patients. For driverml, the *p* < 0.01 was used as the threshold, and for MutSigCV, oncodrivefml, oncodriveclutl, the FDR<0.05 was used as the threshold. The gene that was discovered by two software and more was identified as driver gene of ESCC. Used maftools (Mayakonda et al., 2018) to draw a mutation profile of the driver gene.

### Mutual Exclusion Analysis

We used maftools to analyze gene exclusion and analysis, with a *p* = 0.05 as a threshold.

### Clinical Correlation Analysis

Generally, we divided cancer patients into two groups: young and old by the threshold value of age as 60. To improve the grouping method, we used the kmeans clustering method (https://github.com/dstein64/kmeans1d) to divide the patients into two groups according to age. And the clustering was also based on 60 years old. The samples were divided respectively into two groups by the different clinical characteristics: T stage (T1-T2 VS T3-T4), TNM (S1-S2 VS S3-S3), gender (male VS female), lymph node metastasis (YES VS NO), smoking (YES VS NO), drinking (YES VS NO), tumor grade (G1-G2 VS G3-G4). For the tumor location, the samples were divided into upper, middle, and lower three groups. And because there are only 6 samples of M1, so we do not analyze M stage. Clinical information of all ESCC were in Supplementary Table S3.

### Data Visualization

We used ggscatterstats, ggwithinstats, venn, volcano graph, and histogram to draw related graphs on the hiplot platform (https://hiplot.com.cn/), and used R ggplot2 package to plot other graphs.

### Statics Analysis

The Fisher test was used to analyze the gene mutation with clinical characteristics, and the *t*-test was used to analyze the correlation between the gene mutation with numerical variables, such as age, aTMB, fTMB etc.

For survival analysis, we used the survminer of the R package to analyze the survival differences between groups. For numerical variables, we use surv_cutpoint to determine the best cut point.

### Cell Culture Conditions

The ESCC cell lines used in this study were from our lab. All ESCC cell lines were cultured in HyClone™ RPMI-1640 medium (GE Healthcare Life Sciences, HyClone Laboratories, Logan, UT, United States) with 10% fetal bovine serum (FBS; Gibco; Thermo Fisher Scientific, Inc., Waltham, MA, USA) at 37°C in a 5% CO_2_ incubator. Replace the culture medium according to the cell status and subculture cells when the density of cells increases to about 80–90%.

### Knockdown of KMT2D in ESCC Lines

For the knockdown of KMT2D, we used the interfering RNAs (siRNAs) containing the sequence: 5′-GCA​CCA​TCA​TTC​GGA​ACG​A-3’ (KMT2D-si1) and 5′- CCA​GTA​CTT​TCG​CTT​CGA​A -3’ (KMT2D-si2). Cells at the logarithmic growth phase were transfected with siRNA using riboFECTTM CP Reagent (RIBOBIO, Guangzhou, China) according to the company’s recommendation. The efficiency of knockdown was determined by real time-PCR.

### RNA Extraction and Real Time PCR

Total RNA of ESCC cells was isolated by using RNAiso plus (Takara, Dalian, China). Complementary DNA (cDNA) was synthesized from 2 μg of total RNA using a PrimeScript^®^ RT reagent Kit with gDNA Eraser (Takara). TB Green^®^ Premix Ex Taq^®^ II kit (Takara) was used in real time PCR. All real time PCR reactions were performed in triplicate with an Applied Biosystems Step One Plus (ABI, Foster City, CA, United States). The relative expression of target genes was determined by normalization to GAPDH expression and calculated using the 2^−ΔΔCt^ formula. The primer sequences of GAPDH and KMT2D used in this study were listed in Supplementary Table S7.

### Cell Invasion and Migration Assay

The invasion and migration ability of cells were detected using transwell plates (8 μm, Corning, Inc.). For the migration assays, 50,000 to 80,000 cells/well were inoculated into the upper compartment of the transwell plate with serum-free culture. The lower part of the chamber was filled with culture fluid containing 10% FBS. The upper surface cells are removed after 24 h or 48 h. The cells passing through the membrane are fixed with 4% formaldehyde and stained with 0.1% crystal violet. Random five fields were chosen to count the number of transmigrated cells. For the invasion assays, the upper chambers were precoated with 100 μl Matrigel (1: 8 mixed with FBS-free media; BD Biosciences, Heidelberg, Germany) and proceeded as the same as described above.

### Cell Proliferation Assay

In total, 5 × 10^3^ transfected cells were seeded into each well of a 96-well plate in a final volume of 200 µl. After cultured for 24, 48, 72, 96 h, 20 μl CCK8 solution was added into each well, then the cells were incubated at 37°C for 30 min. The relative number of surviving cells in each group was measured by a spectrophotometer at 490 nm.

### Colony Formation Assay

Cells were seeded in a 6-well plate at a density of 1,000 or 1,500 cells/well and incubated for 10 days at 37°C with 5% CO_2_. After PBS cleaning, The cells were fixed with 4% polyformaldehyde for 30 min and stained with 1% crystal violet for 30 min. The numbers of colonies containing more than 50 cells were counted under the microscope.

## Results

### ESCC Cohort and Samples

**FIGURE 1:**
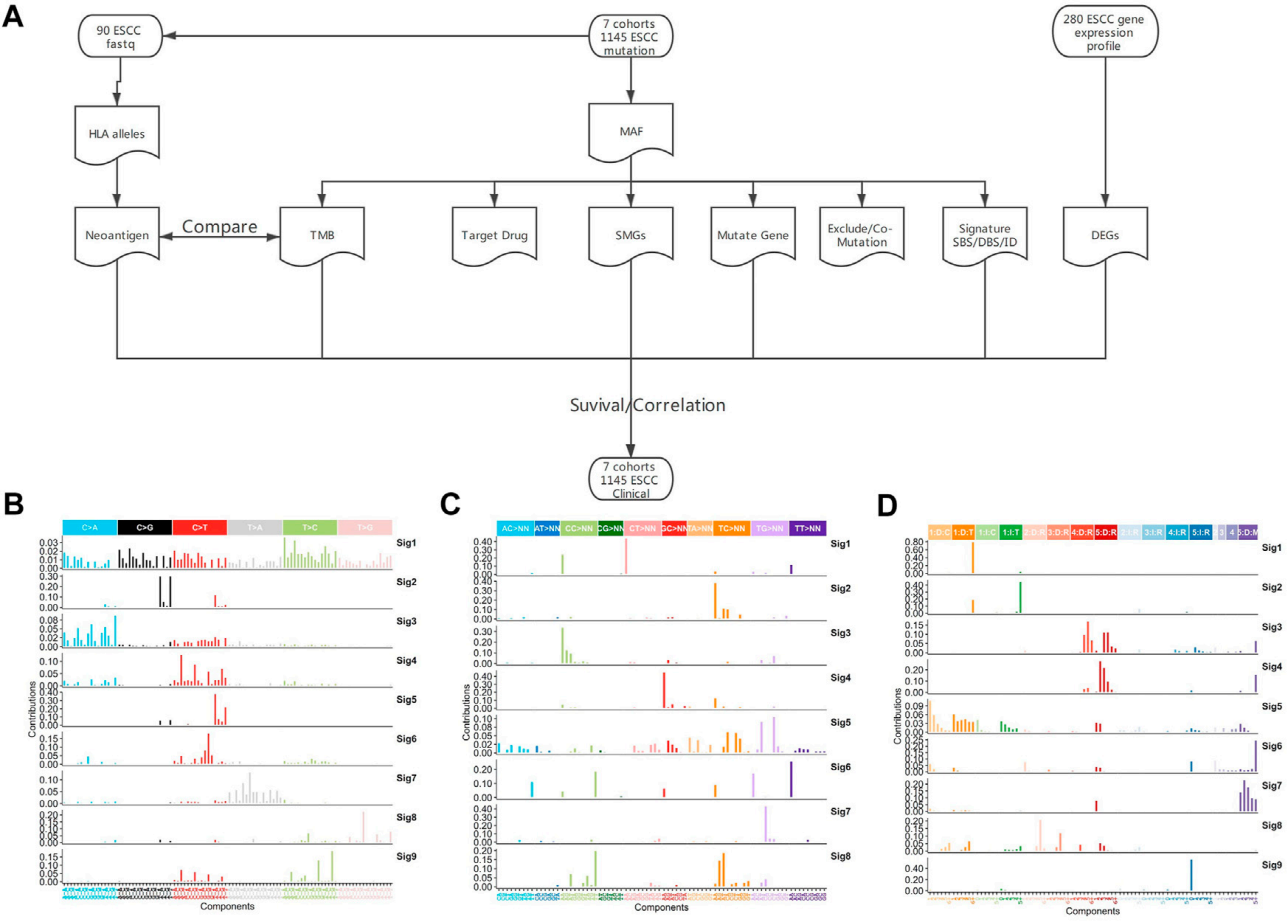
Analysis pipeline and mutation signature of whole genome region in ESCC. **(A)** Analysis pipeline, **(B)** SBS signature of WGS, **(C)** DBS signature of WGS, **(D)** ID signature of WGS.

Dozens of research cohorts have been published since 2014 (Supplementary Table S1) (Liu et al., 2017; Yu et al., 2019; Dutta et al., 2020; He et al., 2020; Mangalaparthi et al., 2020; Song et al., 2014; Cao et al., 2015; Zhang et al., 2015; Hu et al., 2016; Qin et al., 2016; Sawada et al., 2016; Chang et al., 2017; Chen et al., 2017; Dai et al., 2017; Du et al., 2017; Dai et al., 2018; Yan et al., 2019; Cui et al., 2020; Liu et al., 2020; Nature, 2017; Lin et al., 2014). In this study, we integrated and re-analyzed the 7 large cohorts including a total of 1,145 samples (Table 1) (Lin et al., 2014; Song et al., 2014; Zhang et al., 2015; Sawada et al., 2016; Chang et al., 2017; Nature, 2017; Cui et al., 2020). The whole analysis pipeline was shown in Figure 1A. There were 508 WGS samples and 637 WES samples a total of 149,085 mutations in the coding region (Supplementary Table S2). The median of ESCC tumor mutation burden (TMB) of nonsilent mutation in the coding region was located between Uterine Corpus Endometrial Carcinoma (UCES) and Liver hepatocellular carcinoma (LIHC), and was lower than Esophageal adenocarcinoma (EAC) (Supplementary Figure S1).

**TABLE 1:** ESCC cohort and samples.

Year	ESCC sample number	Journal
2014	88	*Nature*
2014	113	*Nature Genetics*
2015	104	*American Journal of Human Genetics*
2016	144	*Gastroenterology*
2017	96	*Nature*
2017	92	*NATURE COMMUNICATIONS*
2020	508	*Cell Research*

### Mutational Signatures in ESCC

**FIGURE 2:**
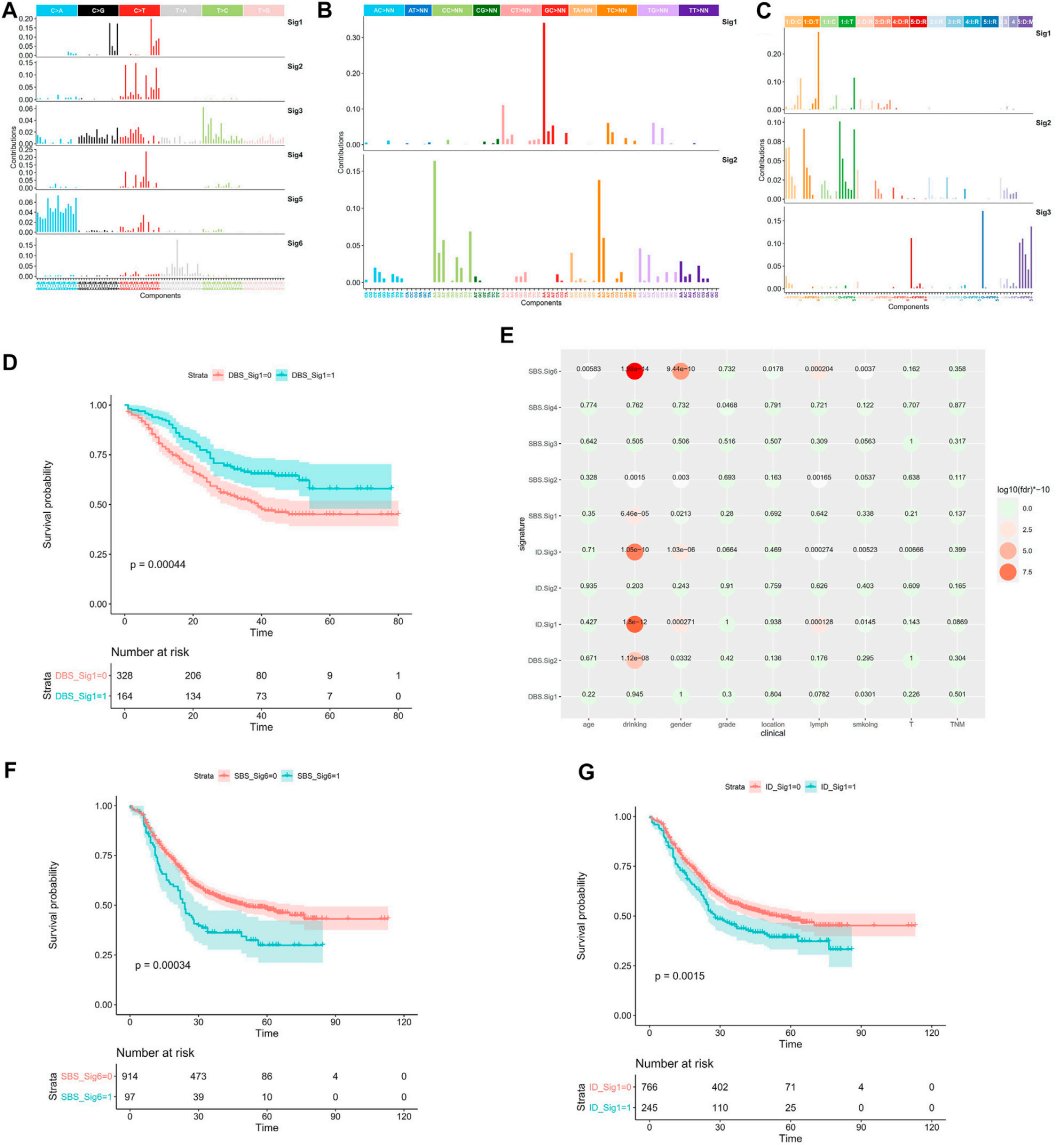
Mutation signature of coding region in ESCC. **(A)** SBS signatures of WES, **(B)** DBS signatures of WES, **(C)** ID signatures of WES, **(D)** WGS-DBS-S1 survival analysis, **(E)** WES signature correlation with clinical characteristics, **(F)** WES-SBS-S6 survival analysis, **(G)** WES-ID-S1 survival analysis. In the survival analysis, red line represents the ESCC patients enrichment in the signature, the green line represents ESCC patients without enrichment in the signature.

The previous analyses of ESCC mutation signatures of ESCC were all based on single base substitution (SBS). Here, we re-analyzed the SBS, double-base substitution (DBS), and small insertions and deletion (ID) signatures separately in both the coding regions of 1,145 ESCC samples and the whole genome of 508 ESCC using sigflow (Wang et al., 2020a). Total of 8 SBS, 9 DBS, 9 ID signatures were identified in the whole genome region (Figures 1B–D). 6 SBS and 2 DBS, and 3 ID signatures in the coding regions (Figures 2A–C). Annotated with the COSMIC signatures, we found that the signatures in the coding region (Supplementary Table S4) and those in whole genome region (Supplementary Table S6) are similar in some aspects. A larger number of signatures were identified in our study. Besides that, two new signatures that have not been reported before were discovered in the whole genome region. One was WGS-SBS-S3 related to possibly damage by reactive oxygen species and the other was WGS-DBS-S1 related to prior chemotherapy treatment with platinum drugs. WGS-DBS-S1were correlated with the overall survival (OS) rates (Figure 2D, *p =* 0.00044). Next, we analyzed the correlation between signatures of coding regions and clinical characteristics (Figure 2E, Supplementary Table S5) or the OS rates. We found that most of them were related to drinking, smoking, gender and lymph metastasis (FDR<0.05). WES-SBS-S6 (Figure 2F, *p =* 0.00044) and WES-ID-Sig1 (Figure 2G, *p =* 0.00044) were correlated with the OS rates.

### Tumor Mutation Burden in ESCC

**FIGURE 3:**
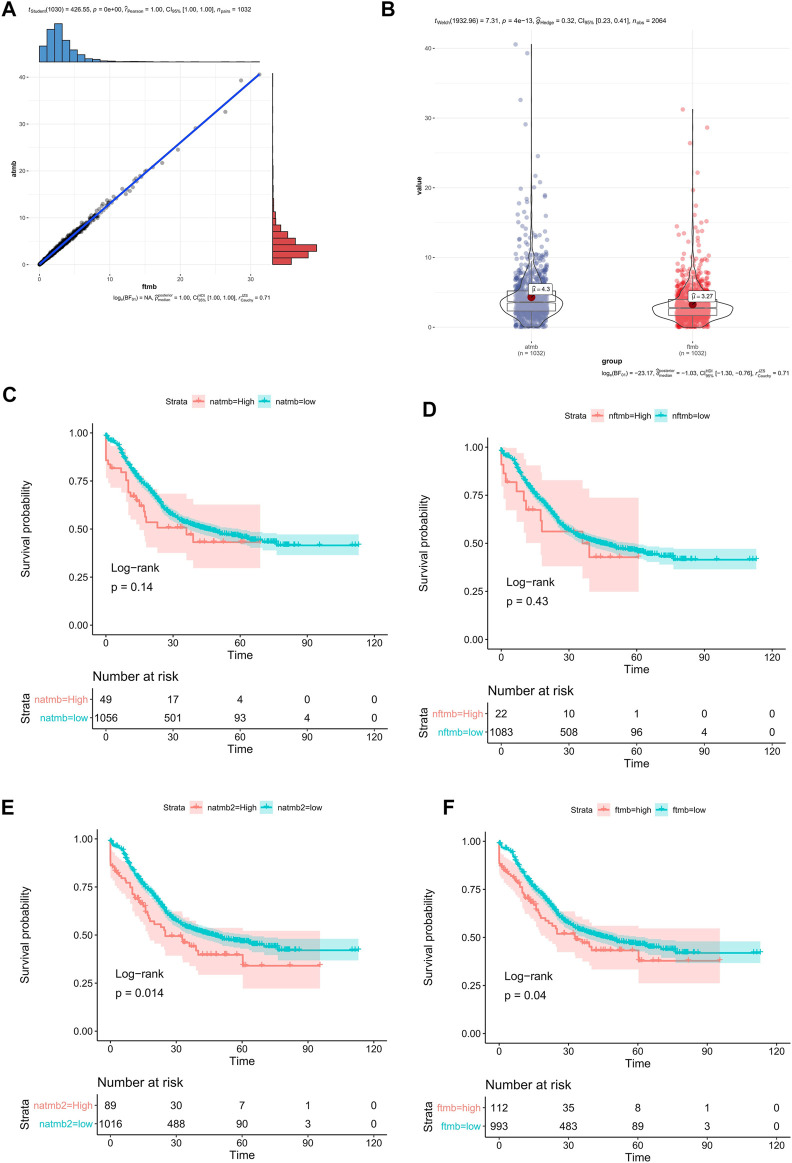
TMB in ESCC. **(A)** the correlation between aTMB and fTMB, **(B)** compared the mean value between aTMB and fTMB, **(C)** Survival analysis of aTMB (high aTMB >10), **(D)** Survival analysis of fTMB (high fTMB >10), **(E)** Survival analysis of aTMB (high aTMB >8.0), **(F)** Survival analysis of fTMB (high fTMB >5.7).

TMB is an important cancer immunotherapy biomarker (Boumber, 2018a; Chan et al., 2019; Jardim et al., 2020). Adult and pediatric patients with high TMB (≥10 mutations/megabase) were approval for pembrolizumab by FDA (Subbiah et al., 2020). Currently, there is not a standard way to calculate TMB. Some studies excluded synonymous mutations (Zehir et al., 2017), while some studies include synonymous mutations (Xu et al., 2019). In this study, we defined aTMB which includes synonymous mutations and fTMB which excludes synonymous mutations. The average value of aTMB was 4.30 in the 1145 ESCC patients, and the median value of aTMB was 3.58 in the 1145 ESCC patients. The average value of fTMB was 3.27 in the 1145 ESCC patients, and the median value of aTMB was 2.75 in the 1145 ESCC patients. The consistency between aTMB and fTMB in ESCC was significant (Figure 3A,R = 1, *p =* 0), but the values calculated by the two methods are significantly different (Figure 3B, *p =* 4.13e-13). Thus, we speculated that the calculation method needed to be considered when using a specific threshold.

We compared different group OS rates using different TMB values. If grouping patients with the TMB value 10 (Cui et al., 2020), there was no significant difference between the high TMB group and the low TMB group regardless of using aTMB (Figure 3C, *p =* 0.14) or fTMB (Figure 3D, *p =* 0.43) method. However, using survminer (Kassambara et al., 2017), the optimal grouping point was automatically selected by the surv_cutpoint method. Using the aTMB value 8.0 as the threshold, we found that there was a significant difference OS rates between the high and low TMB groups (Figure 3E, *p =* 0.014). There was also significant difference between the high and low fTMB groups (fTMB >5.7, Figure 3F, *p =* 0.04). Therefore, different analysis methods require the corresponding TMB threshold values. In addition, the commonly defined high-TMB threshold value should not be applied in different cancer types. For ESCC, we thought the aTMB as 8.0 was better than 10. And fTMB as 5.7 was recommended for the similar study. However, the accurate value of the high TMB threshold suitable for clinical grouping requires further validation.

### High Heterogeneity of Neoantigen in ESCC

Neoantigens play an important role in tumor immunotherapy. We analyzed the neoantigens of 90 WES samples (Zhang et al., 2015) with pvactools (Hundal et al., 2020) for obtaining fastq data. 2 new neoantigen indicators were defined as follows. One was the tumor neoantigen burden (TNB), which was the number of neoantigen in the sample. The other was the tumor neoantigen score (TNS), which was the score of neoantigen in the sample (See methods).

**FIGURE 4:**
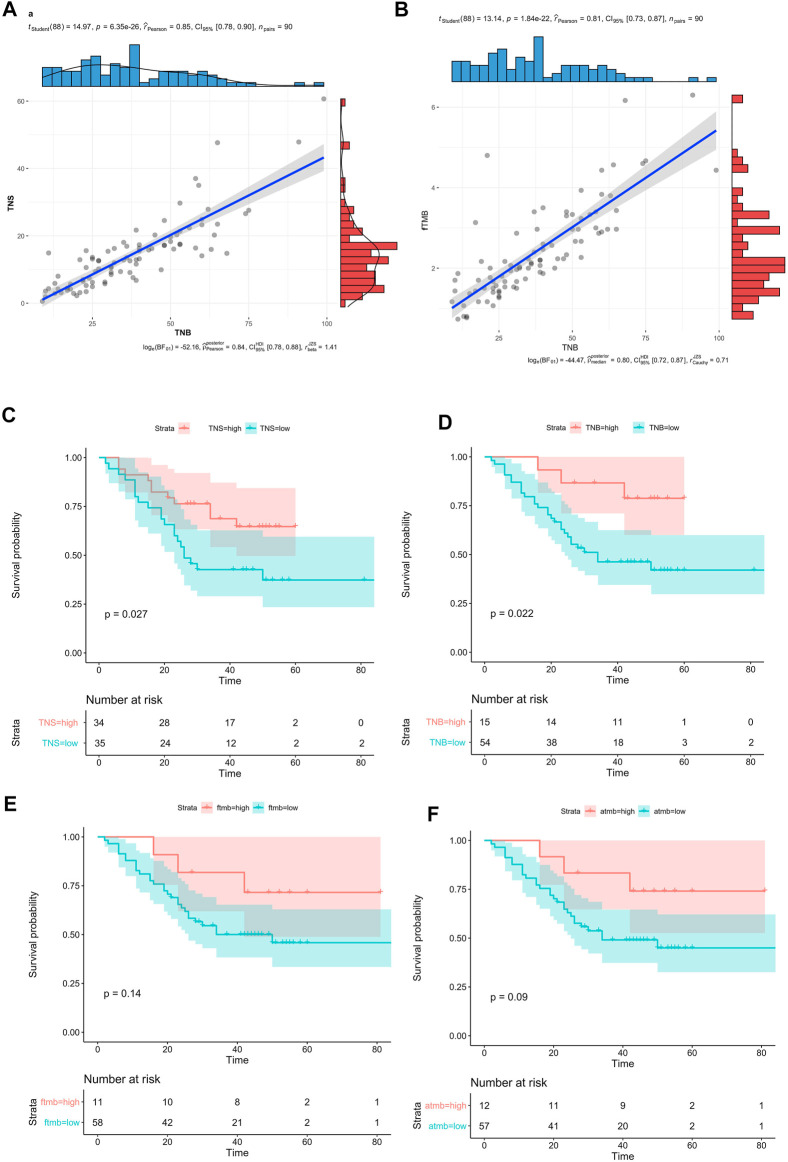
Neoantigen in ESCC. **(A)** The correlation bewtten TNB and TNS, **(B)** Compared the mean value between TNB and fTMB, **(C)** Survival analysis of TNS (high TNS >2.2), **(D)** Survival analysis of TNB (high TNB>2.5), **(E)** Survival analysis of fTMB (high fTMB >1.6), **(F)** Survival analysis aTMB (high aTMB >1.8).

The correlation between TNS and TMB was very high (Figure 4A, R = 0.85, *p =* 6.35e-26), and the correlation between TNB and fTMB was also high (Figure 4B, R = 0.81, *p =* 1.84e-22). Since there were no mutation hotspots in ESCC, the highest mutation frequency was TP53:p.R282W (2.41%), so the neoantigens produced by patients are different. The highest frequency of the neoantigen peptides can only be found in 3 patients. Only 2 of this neoantigen accounted for 0.6% of all neoantigens. 60 neoantigens appeared in 2 patients accounted for 1.8%. The proportion of neoantigens that appeared in one patient was 97.6%. Therefore, we thought that the effective cancer vaccine is hard to be developed for ESCC based on our results.

The 90 patients were divided into 2 groups based on the TNB or the TNS, by the surv_cutpoint method in survminer. There were significant differences between the high and low groups (TNS>2.2, *p =* 0.027, Figure 4C; TNB>2.5, *p =* 0.022, Figure 4D). Compared with the TNB high group, the high group of TNS had more patients, the ratio of the high-low group is to 34:35, and the ratio is 15:54 in the TNB.

Compared with neoantigen, 90 patients were divided into 2 groups based on the fTMB (high fTMB>1.6) or aTMB (high aTMB>1.8), by the surv_cutpoint method in survminer. There was no significant difference between the high and low groups (Figure 4E, *p =* 0.14, Figure 4F, *p =* 0.09). Compared with the OS, neoantigen was a better biomarker than TMB. And the TNS was a suitable factor for neoantigen calculation compared with the TNB.

### Potential Targeted Therapy Drugs in ESCC

At present, limited targeted drugs have been approved for ESCC. In order to find potential targeted drugs for ESCC, we searched the targeted drug databases OncoKB (Chakravarty et al., 2017) and Civic (Griffith et al., 2017). Currently, there were no special targeted drugs for ESCC in the OncoKB database. In the Civic database, the targeted drugs for ECA and ESCC were different, but the highest evidence level was only B for ESCC. Recently, some studies have begun to treat different cancer patients with specific targeted drugs based on the same gene mutation position (Gambacorti‐Passerini et al., 2018). Our study uses the OncoKB and Civic databases for analysis of targeted drugs for particular gene mutation in ESCC. We found that there are only drugs targeting only TP53 and PIK3CA gene mutations (Supplementary Figure S4). Among them, 135 patients, accounting for 11.8%, can benefit from the drug AMGMDS3 targeting TP53 gene (Supplementary Figure S4). From the data of the two databases, patients with ESCC can benefit little from the existing targeted drugs. More research is required to find new targeted drugs in the future.

### Significant Mutated Genes in ESCC

**FIGURE 5:**
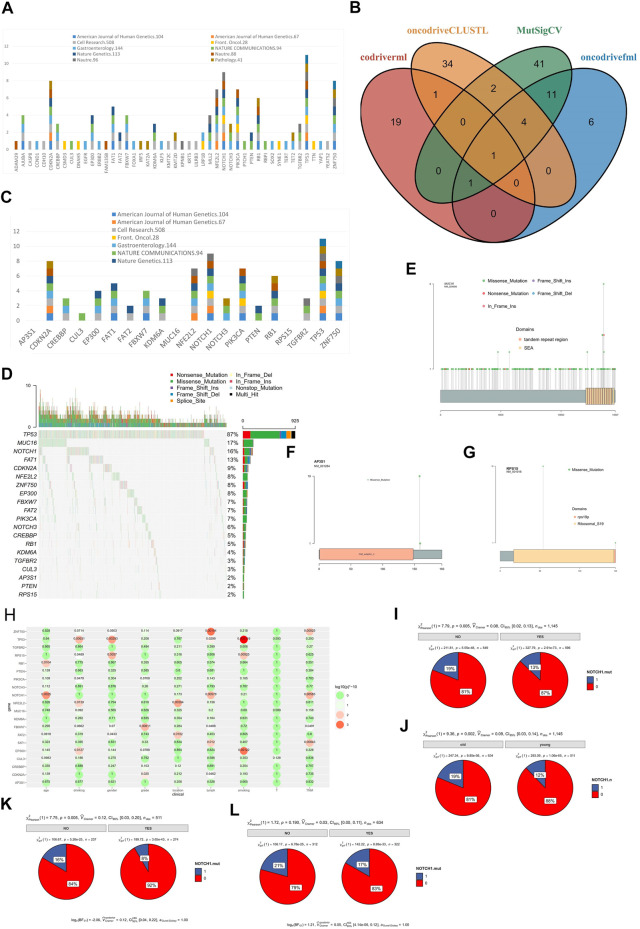
SMGs in ESCC. **(A)** SMGs of ESCC in previous studies, **(B)** SMGs of ESCC identified by 4 software, **(C)** The distribution of SMGs of ESCC identified by 4 software in previous studies, **(D)** Mutation profile of SMGs in ESCC, **(E)** *MUC16* mutation in ESCC, **(F)** *AP3S1* mutation in ESCC, **(G)** *RPS15* mutation in ESCC, **(H)** The correlation between SMGs of ESCC and clinical characteristics, **(I)** The correlation between *NOTCH1* mutation and lymph node metastasis of ESCC, **(J)** The correlation between NOTCH1 mutation and different age group of ESCC patients (young and old), **(K)** The correlation between NOTCH1 mutation and lymph node metastasis of young ESCC patients (age<60), (L) The correlation between NOTCH1 and lymph node metastasis of old ESCC patients (age >= 60).

We re-analyzed more than 20 sequencing datasets (Liu et al., 2017; Yu et al., 2019; Dutta et al., 2020; He et al., 2020; Mangalaparthi et al., 2020; Song et al., 2014; Cao et al., 2015; Zhang et al., 2015; Hu et al., 2016; Qin et al., 2016; Sawada et al., 2016; Chang et al., 2017; Chen et al., 2017; Dai et al., 2017; Du et al., 2017; Dai et al., 2018; Yan et al., 2019; Cui et al., 2020; Liu et al., 2020; Nature, 2017; Lin et al., 2014) of ESCC and found that only 11 studies included the driver gene analyses (Supplementary Table S1). Seven of them used MutSigCV for analysis (Lawrence et al., 2013), dNdScv was used in one study (Martincorena et al., 2017), and MutSigCV and oncodriveFML were applied together in another study (Mularoni et al., 2016). A total of 47 driver genes were found in 11 published studies. Among them, 7 genes (TP53 (11), NOTCH1 (9), CDKN2A (8), ZNF750 (8), NFE2L2 (7), PIK3CA (7), RB1 (6)) were considered driver genes in more than 5 studies and 26 genes were considered driver genes in only one study (Figure 5A). Previous studies were limited because of using fewer analysis tools or the relatively small sample size. Therefore, the reported driver genes identified by previous studies is limited.

To identify the driver genes of ESCC in a more accurate and comprehensive way, four software were applied in our study: MutSigCV, driverml (Han et al., 2019), OncodriveFML, and OncodriveCLUSTL (Arnedo-Pac et al., 2019). A total of 20 driver candidate genes were identified (Figure 5B, Figure 5C). Among them, *TP53* was a driver gene recognized by all software. *EP300*, *FAT1*, *FBXW7*, *PIK3CA*, *ZNF750*, and *AP3S1* were identified as driver genes in three software. Among the 20 driver genes, 14 genes were reported in three or more studies, *FAT2* and *PTEN* were reported as driver genes in only 2 studies, and *CUL3* was reported as driver gene in only 1 study. *AP3S1*, *MUC16*, *RPS15* had not been reported as a driver gene (Figure 5C). *AP3S1*, *MUC16*, and *RPS15* were three newly discovered driver genes. The *MUC16* had been considered as a driver gene in the pan-cancer driver gene research (Martínez-Jiménez et al., 2020), the mutation frequency of *MUC16* in ESCC was high (17%, Figure 5D), and there was not hotspot mutation in the *MUC16* (Figure 5E). while *AP3S1* and *RPS15* had not been reported as driver genes before, and there were hotspot mutations in the *AP3S1* (Figure 5F) and *RPS15* (Figure 5G) in the ESCC, the mutation frequency of *AP3S1* and *RPS15* were low in the TCGA, and there was not hotspot mutation in the TCGA (Supplementary Figures S2, S3).

Next, we analyzed the correlation between driver gene and clinical characteristics (Figure 5H). The results showed that some genes were related to multiple clinical characteristics, such as *ZNF750* related to lymph node metastasis and TNM stage. *NOTCH1* mutated were related lymph node metastasis (*p =* 0.005, Figure 5I) and related to age (*p =* 0.002, Figure 5J). Moreover, we found that *NOTCH1* mutated was related to young (*p =* 0.005, Figure 5K), not to old (*p =* 0.190, Figure 5L).

We analyzed the mutual exclusion and co-mutation of all mutant genes (Supplementary Figure S5), and found that *ZNF750* and *CDKN2A* was mutually exclusive (*p =* 0.004809937). A total of 52 pairs of genes have been found to have co-mutations, the most significant of which are TTN and lFSIP2 (*p =* 2.94E-05).

### Correlation Between Gene Mutation and Lymph Node Metastasis

**FIGURE 6:**
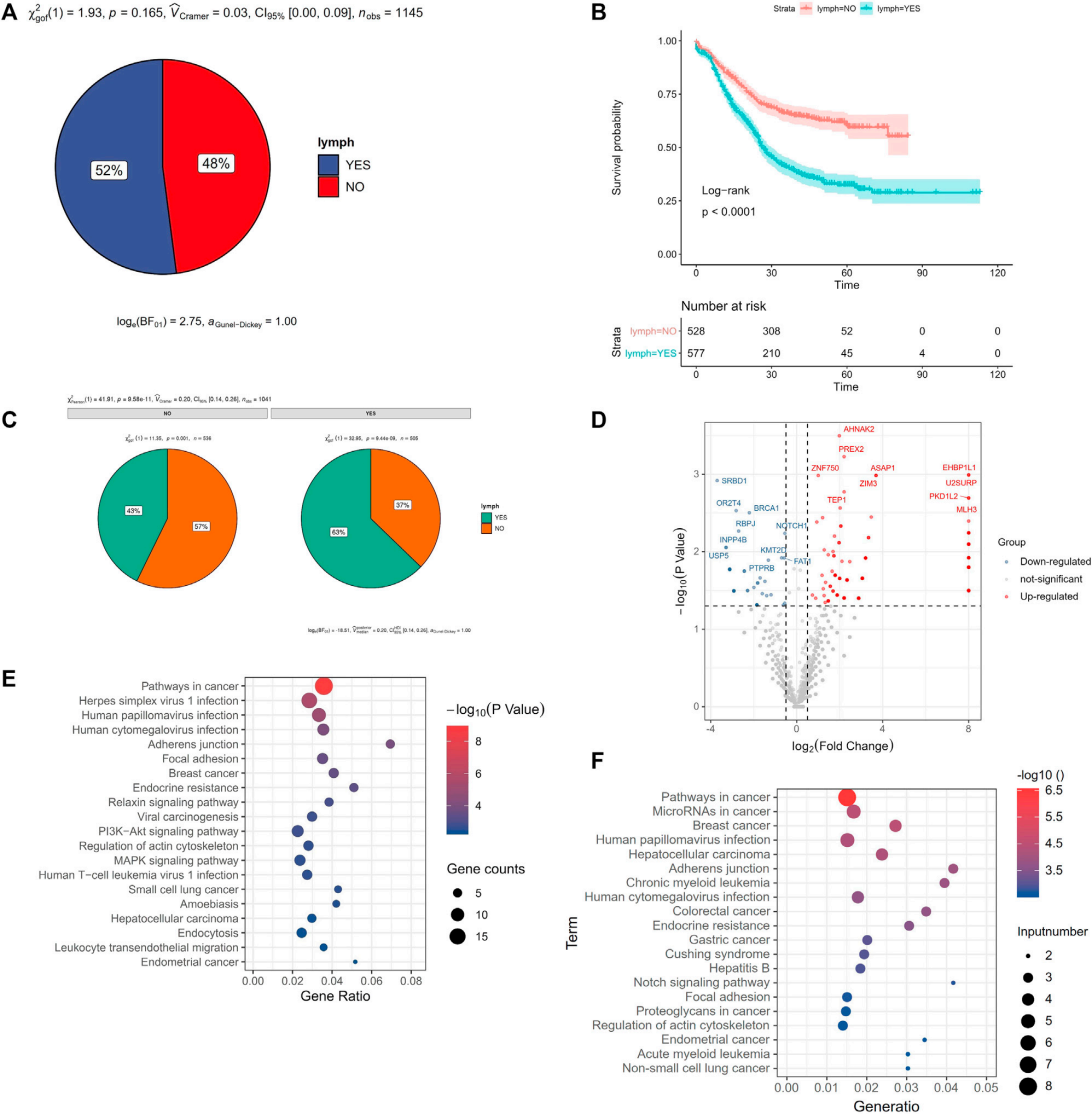
Gene mutation with lymph node metastasis. **(A)** the frequency of lymph node metastasis in ESCC, **(B)** Survival analysis of lymph node metastasis in ESCC, **(C)** smoking related with lymph node metastasis in ESCC, **(D)** volcano plot of the correlation gene mutation with lymph node metastasis, *x*-axis was the log_2_ (the ratio of mutated rate of lymph node metastasis group and the mutated rate of without lymph node metastasis group), and *y*-axis was *P* (the correlation gene mutation with lymph node metastasis), **(E)** KEGG enrichment pathway of the higher mutation gene of lymph node metastasis compared with no lymph node metastasis, **(F)** KEGG enrichment pathway of the lower mutation gene of lymph node metastasis compared with no lymph node metastasis.

Most cancer death was originally caused by metastasis. Tumor metastasis was mainly included lymph node metastasis and blood metastasis. In our study, there were 52% ESCC patients with lymph node metastasis (Figure 6A). And the survival time of ESCC patients with lymph node metastasis was lower than that of ESCC patients without lymph node metastasis (*p* < 0.0001, Figure 6B). We found that the drinking history, smoking history, T stage, tumor location and tumor grade were related to lymph node metastasis (*p* < 0.05). It was worth noting that among these clinical characteristics, tobacco consumption largely promoted the increase in the frequency of lymph node metastasis. Compared with non-smoking patients, the frequency of lymph node metastasis was 43%, rising to 63% (*p =* 9.58E-11, Figure 6C).

There were 237 mutated genes related with lymph node metastasis (*p* < 0.05). Among them, 188 genes showed higher mutation rate of lymph node metastasis than no lymph node metastasis group, called “higher mutated genes (HMG)”. These gene mutations may promote lymph node metastasis. While 49 genes showed lower mutation rate in lymph node metastasis group than that in no lymph node metastasis group, called “lower mutated gene (LMG)” (Figure 6D). And these gene mutations may prevent lymph node metastasis. We used kobas (Xie et al., 2011) respectively to do KEGG pathway enrichment analysis of these two types of genes. We found that the Pathways in cancer, Herpes simplex virus 1 infection, Human papillomavirus infection were the top 3 pathways in enrichment of those LHG (Figure 6E). And pathways in cancer, MicroRNAs in cancer, Breast cancer were the top 3 pathways of those LWG (Figure 6F).

### KMT2D Plays a Tumor Suppressor Role in ESCC

**FIGURE 7:**
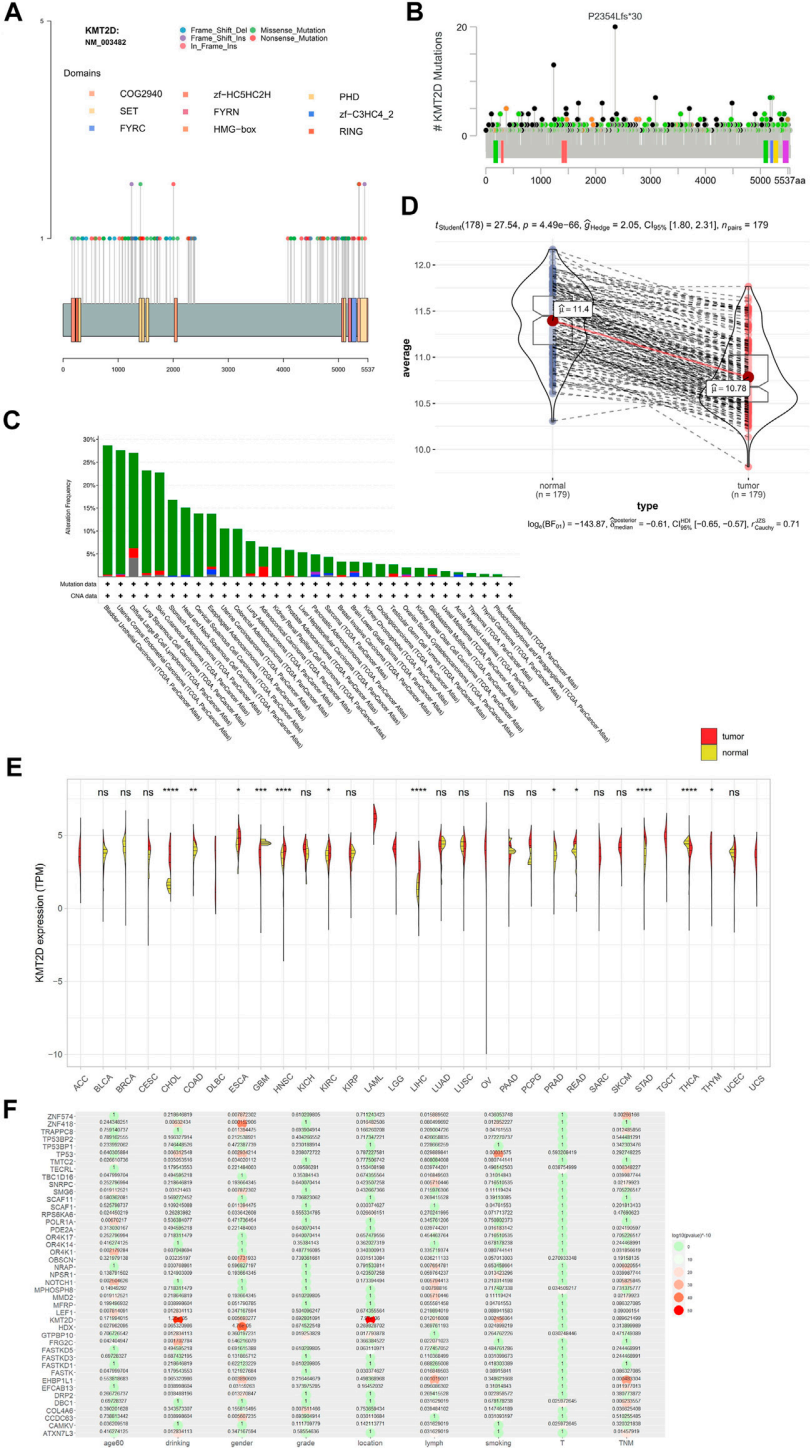
*KMT2D* mutation, *KMT2D* expression and the correlation between *KMT2D* mutation and clinical characteristics. **(A)** *KMT2D* mutation in ESCC, **(B)** *KMT2D* mutation in TCGA, **(C)** mutation and CNV of *KMT2D* in TCGA cancer, **(D)** the expression of *KMT2D* in tumor and normal of ESCC in the GSE53625, **(E)** expression of *KMT2D* in TCGA cancer, **(F)** the correlation of gene mutation with clinical characteristics.

**FIGURE 8:**
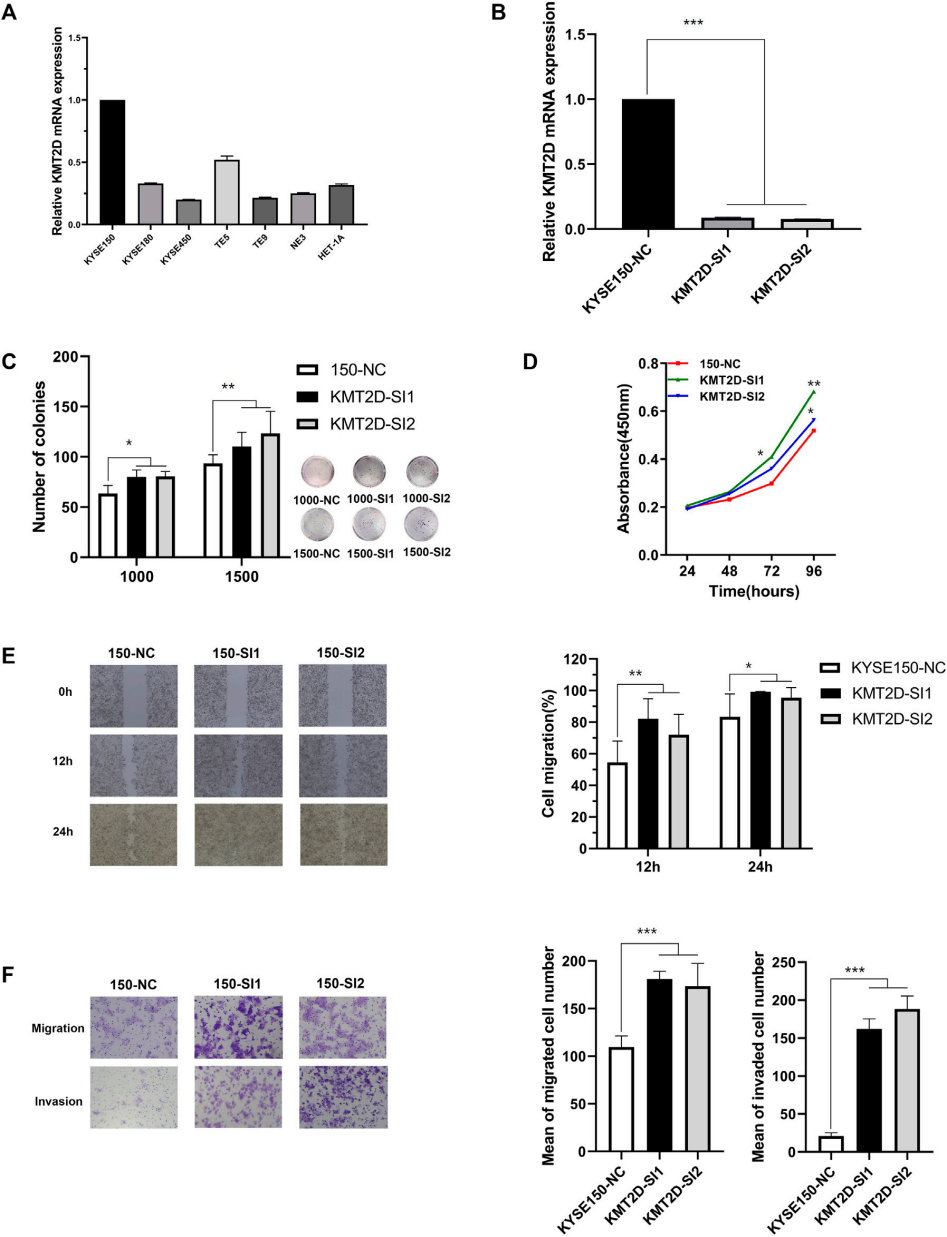
The effect of *KMT2D* in KYSE150 cell line. **(A)** The mRNA expression of *KMT2D* in normal esophageal epithelial cell lines and ESCC cell lines detected by q-RTPCR, **(B)** The knockdown efficiency of *KMT2D* in KYSE150 cell line was verified by q-RTPCR, **(C)** *KMT2D* knockdown promoted the ability of colony formation in KYSE150 cell line compared to the control, **(D)** *KMT2D* knockdown promoted the ability of proliferation in KYSE150 cell line compared to the control, **(E)** *KMT2D* knockdown promoted the ability of migration in KYSE150 cell line compared to the control, **(F)** *KMT2D* knockdown promoted the ability of migration and invasion in KYSE150 cell line compared to the control. All data are presented as the mean ± standard deviation of the three in-dependent experiments.

The frequency of *KMT2D* mutation was 15.02% (top 3 of all mutated gene), and there was no hotspot mutation in this gene mutation in ESCC (Figure 7A) and other cancer (Figure 7B). The *KMT2D* mutation frequency was highest in BLCA closed to 30% (Figure 7C). The expression of *KMT2D* in tumor was lower than that in normal tissues in the GSE53625 (Figure 7D). The different expression pattern between normal and tumor tissues existed in many cancer types in the TCGA data (Figure 7E). We analyzed the correlation between gene mutation and clinical characteristics (Figure 7F). *KMT2D* mutation was related to multiple clinical characteristics (drinking, location, lymph, gender, and smoking, top1 of the number with gene-related clinical characteristics). To verify the biological role of *KMT2D* in ESCC, we compared the expression levels of *KMT2D* in several cell lines including normal esophageal epithelial cells such as NE3, HET-1A and ESCC cell lines such as KYSE150, KYSE180, KYSE450, TE-5, and TE-9. The expression levels of *KMD2T* in different cell lines were shown in Figure 8A. We selected KYSE150 cell line with a relatively high endogenous level expression for perform knockdown experiments. Knockdown of *KMD2T* in KYSE150 cell line showed a significant decrease (Figure 8B). The results of CCK8 and the colony formation assays showed that the cell proliferation, colony formation ability was significantly improved in *KMT2D* knockdown group compared with the control group (Figures 8C,D). Besides that, the knockdown of *KMT2D* can markedly promote cell invasion and migration of KYSE150 (Figures 8E,F).

## Discussion

Mutation signature has always been an important part of cancer research. The analysis of mutation signature has also evolved from SBS to DBS, ID and other complex mutations (Alexandrov et al., 2020). In this study, for the first time, the SBS and ID of ESCC were analyzed. We identified 8 SBS, 9 DBS and 9 ID signatures in the whole genome region. And We also found 6 SBS,2 DBS, and 3 ID signatures the coding region. Most signatures in WGS and WES were similar in ESCC, for example WES-SBS-S1, WGS-SBS-S1 and WGS-SBS-S5 were correlation with APOBEC. However, in the whole genome region, there were more signatures and new signatures identified. For example, WGS-DBS-S1 correlation with prior chemotherapy treatment with platinum drugs, which cannot be identified in the coding region. The mutation signatures in whole genome region can provide more valuable information compared with coding region. This not only deepens our understanding of the mutational signature of ESCC, but also enriches the number of mutational signatures of ESCC. And we also found two new mutation signatures. These mutation signatures can not only be used for the identification of ESCC, but also can be used as new factors for related machine learning or deep learning.

Using a larger size of samples and various driver gene identify software will be helpful in determining the accurate driver genes of ESCC. In our study, we identified 20 drivers using the mutation data of 1,145 samples by using 4 software and 3 (*AP3S1*, *MUC16*, *RPS15*) of them were discovered for the first time. The mutation frequency of *MUC16* in ESCC was high. *MUC16*was recognized as a driver gene in the pan-cancer driver gene research (Martínez-Jiménez et al., 2020). *MUC16* was reported as an important biomarker for cancer (Felder et al., 2014a; Aithal et al., 2018). According to studies in other cancers (Felder et al., 2014b; Aithal et al., 2018; Yeku et al., 2020; Yu et al., 2020), *MUC16* may also be an important immunotherapy target for ESCC. The mutation frequency of *AP3S1*and *RPS15* were low both in ESCC and TCGA, but hotspot mutation was found in the ESCC. Even with hotspot mutations in the two gene but too low mutation frequency limits their value in clinical applications.

We tried to find better immunotherapy and targeted therapy targets for ESCC according to the results obtained from mutations of multiple cohorts. From the OncoKB and Civic databases, there was no targeted therapy drug for ESCC. Both TMB and neoantigen were new immunotherapy markers (Schumacher and Schreiber, 2015; Lu and Robbins, 2016; Łuksza et al., 2017; Boumber, 2018b; Samstein et al., 2019; Zhou et al., 2020; Yin et al., 2021). In the 7 cohorts, the TMB was analysis in only one cohort (508 ESCC patients, published in 2020 years on Cell Research). In this cohort more than 10 mutations per Mb was as TMB-H. TMB was attracted more and more attention by researchers. In recent years, researchers have carried out some systematic research on related immunotherapy on TMB. As a continuous variable, TMB has no recognized cutoff suitable for all cancers. TMB cutoff values is supposed to be different in various cancer types. For ESCC, there is no relevant research to determine the appropriate TMB cutoff. For ESCC, the conventional value for TMB was slightly higher. It may be more appropriate to use the aTMB as 8, and it was more appropriate to use the fTMB as 5.7. The threshold of fTMB and aTMB were caculated by the surv_cutpoint method of “survminer” R package (Kassambara et al., 2017). This method was widely used in various cancers survival analysis for continuous variable (Zhou et al., 2019; Wang et al., 2020b; Lai et al., 2020; Wu et al., 2020). But we need more research to determine the true suitable threshold of TMB. Only a few somatic mutations will produce peptides that have been properly processed and loaded onto the MHC complex. Generally, the more somatic mutations a tumor has, the more likely it is to form a neoantigen (Coulie et al., 2014; Snyder and Chan, 2015). In the 90 WES ESCC cohort, we found that compared with TMB, neoantigen was related to the OS. In ESCC, compared with TMB, neoantigen may be a better marker, however, due to the limitation of sample size, further research is needed. In the relationship between TMB and prognosis, we observed the opposite phenomenon. High TMB had a poor prognosis in 1,145 samples, but in 90 WES samples, high TMB had a good prognosis. This may be due to the number of samples. Similar phenomena have also been found in lung cancer research (Greillier et al., 2018; Alborelli et al., 2020; Nie et al., 2020). Neoantigen therapy is a highly individualized tumor immunotherapy, different patients often have different neoantigens for immunotherapy (Türeci et al., 2018; Blass and Ott, 2021; Jou et al., 2021). For ESCC, neoantigen presents high heterogeneity. Thus, personalized immunotherapy targeting to neoantigens may be a promising treatment for ESCC patients.

Lymph node metastasis is an essential cause of death in cancer (Chaffer and Weinberg, 2011). We analyzed the gene mutation with different clinical characteristics and focused on analyzing the gene mutations with or without lymph node metastasis. Therefore, the identification of biomarkers in ESCC lymph node metastasis will greatly help ESCC treatment and prognosis. Some gene mutations promoted lymph node metastasis, while other gene mutations inhibited lymph node metastasis. We have identified these two types of genes and performed an enrichment pathway analysis, which could help us to understand the mechanism of lymph node metastasis in ESCC. We found that *NOTCH1* mutations were only associated with lymph node metastasis in young ESCC patients, which may be used for the diagnosis of early lymph node metastasis in ESCC.

We found that *KMT2D* (Lysine methyltransferase 2D) was related to multiple clinical characteristics and this gene had a relatively high mutation frequency. *KMT2D* was a gene of histone methyltransferase that methylates the Lys-4 position of histone H3, and there some research in other cancers (Guo et al., 2013; Toska et al., 2017; Xiong et al., 2018; Li et al., 2019; Sun et al., 2019; Alam et al., 2020; Zheng et al., 2021), but there was still a lack of reports in ESCC (Abudureheman et al., 2018). Knockdown of this gene in ESCC cell lines increased invasion and migration ability. The results indicated that *KMT2D* may plays an important role in ESCC. This gene can be used as a potential target for ESCC treatment.

Our results can deepen the understanding of the mutations of ESCC. Based on the mutation data of large samples, we can identify potential biomarkers of ESCC, especially markers for immunotherapy, and provide new ideas for the diagnosis and treatment of ESCC.

## Data Availability

The original contributions presented in the study are included in the article/Supplementary Material, further inquiries can be directed to the corresponding authors.
