# Supplementary figures and images for: Integrative Genomic Analyses of 1,145 Patient Samples Reveal New Biomarkers in Esophageal Squamous Cell Carcinoma
Source: Front Mol Biosci. 2022 Jan 21;8:792779. doi: 10.3389/fmolb.2021.792779 (PMC8814608; doi:10.3389/fmolb.2021.792779)

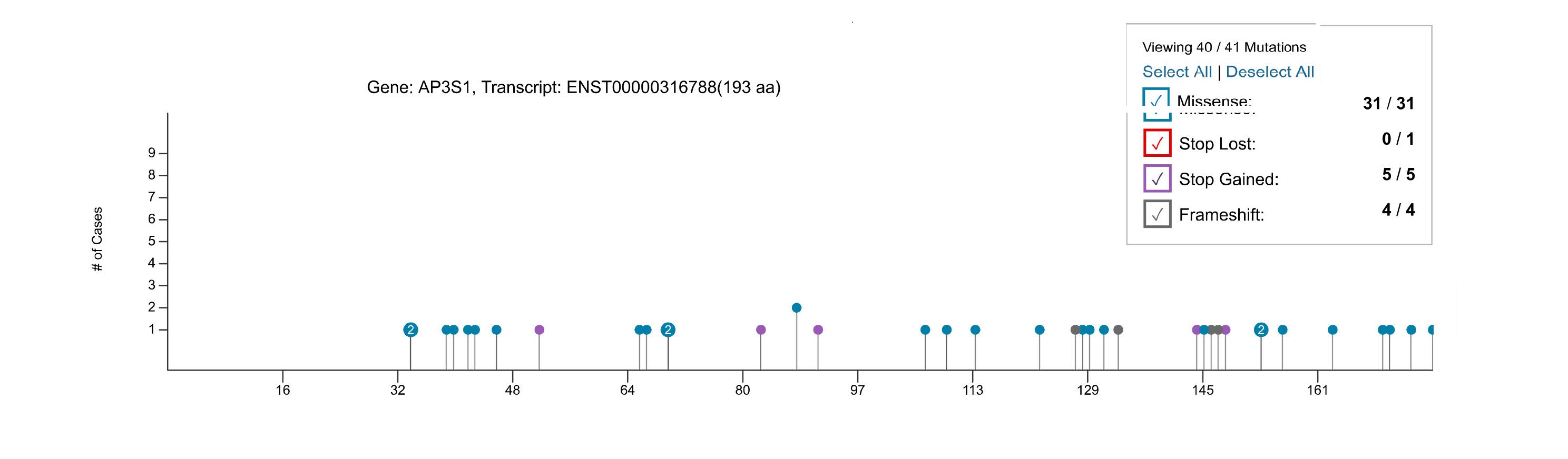

Supplement: Supplementary file 2 [file Image3.JPEG]

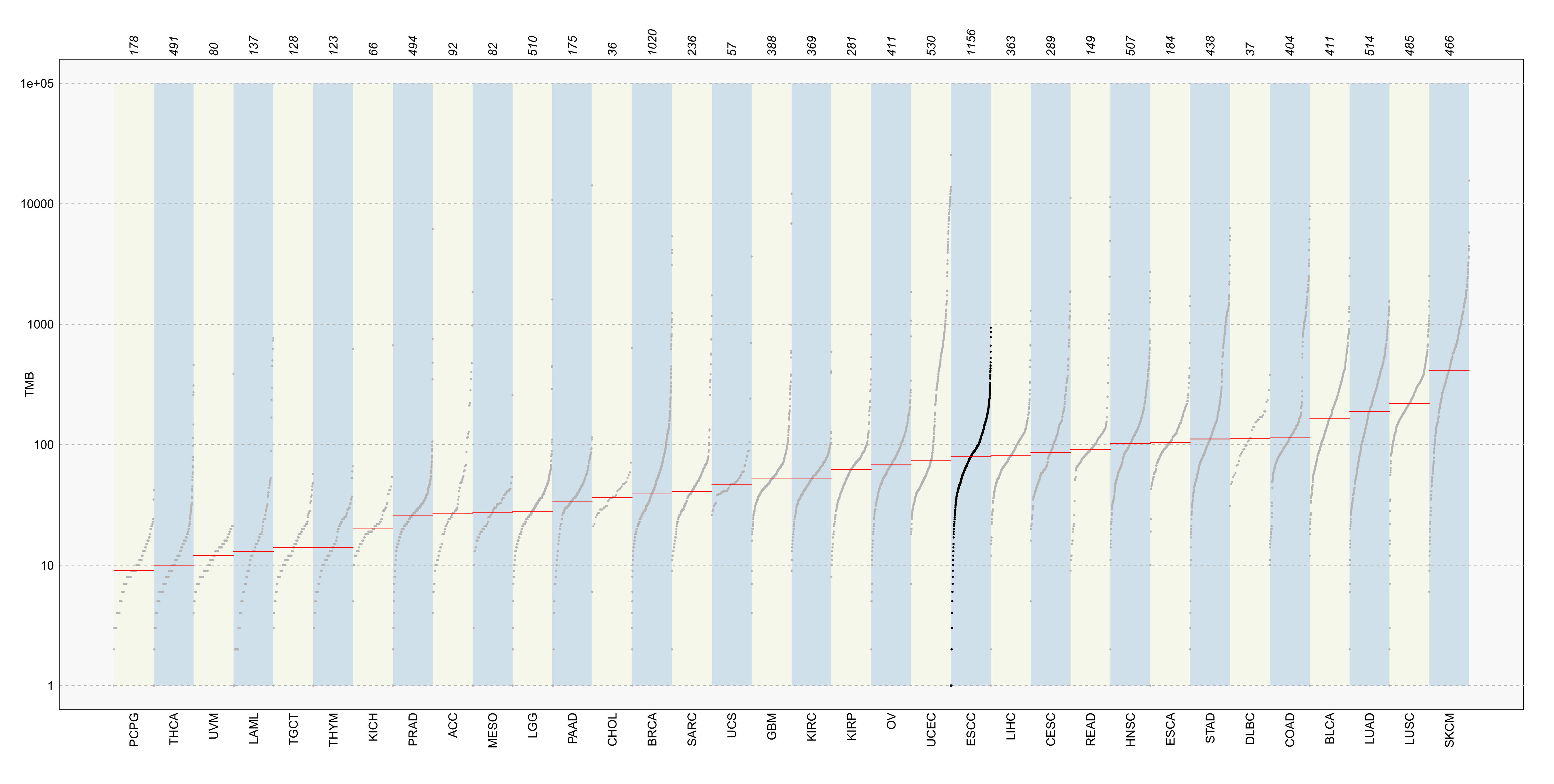

Supplement: Supplementary file 3 [file Image1.JPEG]

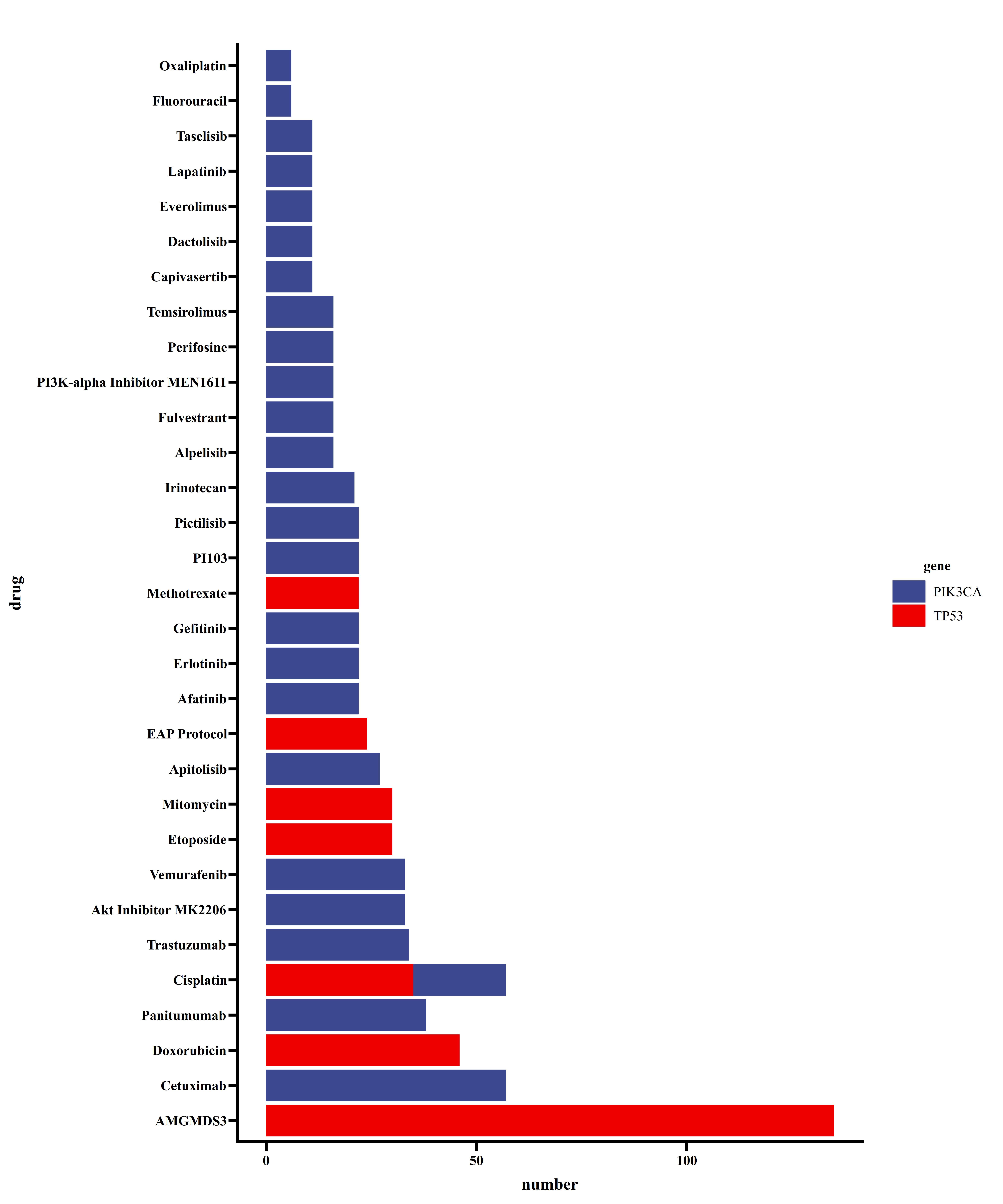

Supplement: Supplementary file 4 [file Image4.JPEG]

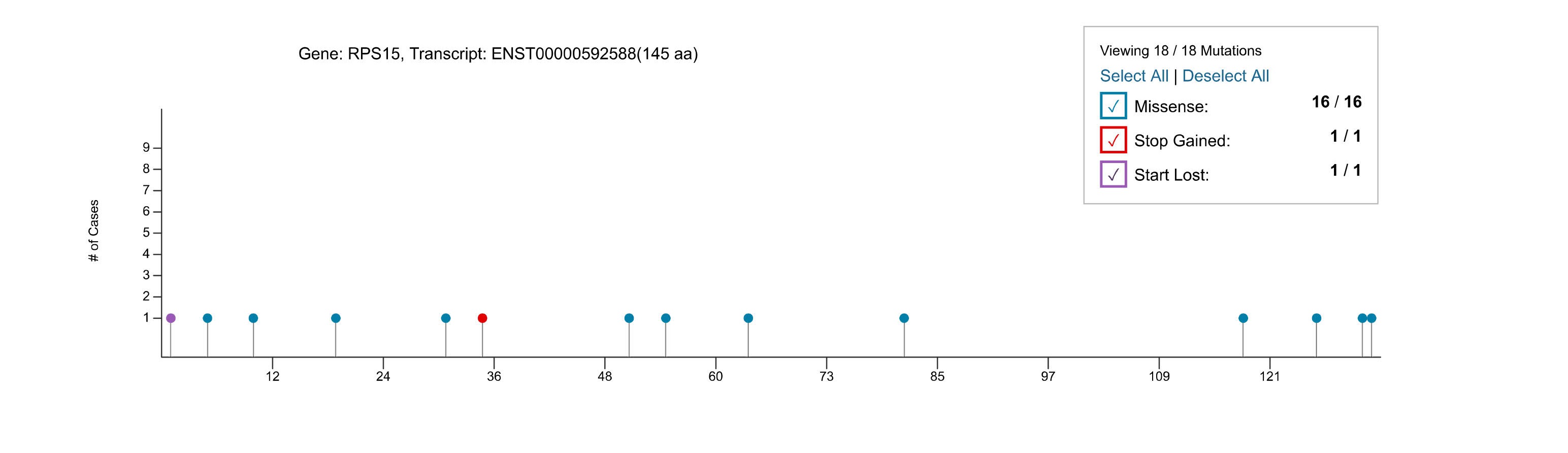

Supplement: Supplementary file 5 [file Image2.JPEG]

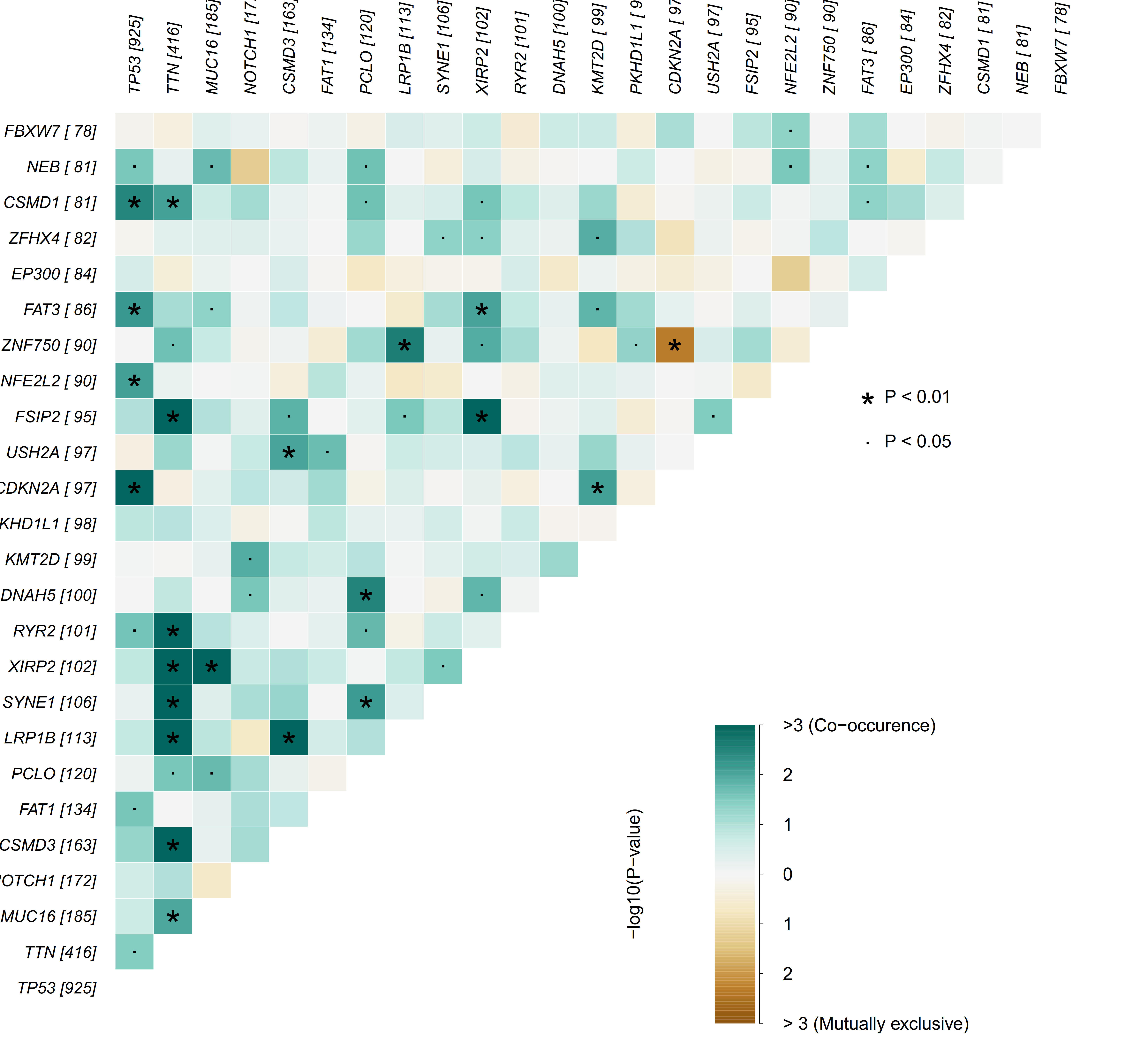

Supplement: Supplementary file 6 [file Image5.JPEG]
